# The London Lancet

**Published:** 1854-04

**Authors:** 


### THE LONDON LANCET.

"After thirty years of a career, more useful and more prosperous than any other medical journal in the world can exhibit, we may point to the remarkable, and we believe, unprecedented fact, that the Lancet is still possessed and directed by the same person that offered the first number to our brethren and to science."

This is the language of the Editor of the *London Lancet* in reviewing the labor of his hands. It is not as modest as it might be but perhaps the veteran Editor has been taught by experience that the conductor of a public journal loses nothing by self-praise. He has, moreover, not a little to felicitate himself upon in the career of his singularly successful journal. It has had a wide circulation, and exercised a beneficent influence upon the medical profession all over the world. But we see no good reason why the Editor, while sounding his own praise, should go out of his way to disparage cotemporary journals. The *Medico-Chirurgical Review* cannot be said in any true sense, to have "dragged on a painful and precarious life," nor the *British and Foreign Review*, to have had "a rapid ephemeral existence." The career of the former, under its original name, was a long and brilliant one,—as long, we believe, and certainly as brilliant and useful as that claimed by its editor for the Lancet; and it has lost nothing of spirit, dignity, or learning by its union with the former. One of the most able journals of medicine ever published in the English language, beyond all question, was the *British and Foreign Medical Review*, and we confess that it is not without some feeling of indignation that we see the nose of Mr. THOMAS WAKLEY, albeit an "M. P. during eighteen years for the Metropolitan Borough of Finsbury," turned up at a journal that numbers among its contributors such men as TODD, BOWMAN, CARPENTER, CHURCHILL, MARCEL, JONES, LAYCOCK, KIRKES, JENNER, WALSHE, PAGET, &c., &c. Mr. WAKLEY has not profited by the example of his immortal countryman, mine *Uncle Toby*, nor made the precious discovery, that the world is wide enough, both for his great serial and its more modest but not less worthy competitors. We, here in the backwoods of America, read them all and esteem them highly, which their editors ought to feel to be the greater compliment, seeing how many journals of our own we have to read and admire.

